# Impact of a web-based module on trainees’ ability to interpret neonatal cranial ultrasound

**DOI:** 10.1186/s12909-020-02400-1

**Published:** 2020-12-03

**Authors:** Nadya Ben Fadel, Sean McAleer

**Affiliations:** 1 Neonatal-Perinatal Medicine program, Children’s Hospital of Eastern Ontario, University of Ottawa, 401 Smyth Road, Ottawa, ON K1H 8L1 Canada; 2 Centre for Medical Education, University of Dundee, Dundee, Scotland

**Keywords:** Neonatal cranial ultrasound, Web-based module, Needs assessment, Evaluation, Satisfaction, Neonatal intensive care unit

## Abstract

**Background:**

Accurate interpretations of neonatal cranial ultrasound (CUS) studies are essential skills for physicians in neonatal intensive care units (NICUs) in order to properly diagnose and manage brain injury. However, these skills are not formally taught to pediatric and neonatal-perinatal medicine (NPM) trainees in Canada. Therefore, our study describes the design, implementation, and evaluation of a new web-based learning (WBL) module that focuses on teaching these skills.

**Methods:**

Trainees’ needs assessment survey, sent to all NPM and pediatrics trainees (*n* = 62), concluded that most of them feel uncomfortable with their ability to interpret CUS, highlighting the need for a new educational intervention. The needs assessment informed the development of the WBL module, which we evaluated using questionnaires and pre-and post-testing methods to measure participants’ satisfaction, knowledge gain, skills development, and behaviour changes. Only trainees rotating through the NICU over 6 months (*n* = 23) were invited to participate in all the evaluation steps. We used the ADDIE instructional design model as a framework for this project.

**Results:**

Respondents were very satisfied with the module, and their baseline knowledge increased significantly after studying and engaging with the module. The post-test score was 76% (*p* < 0.001) compared to the pre-test mean score of 42%. Tests for CUS interpretation skills assessment showed that 49% of pre-test answers were incorrect compared to 8% in the post-test (*p* < 0.001). Seventy-eight percent of trainees (*n* = 18) responded to a survey conducted a year after implementation, and 78% of the respondents (*n* = 14) reported that they still used these skills and shared this knowledge with junior trainees.

**Conclusion:**

A WBL module for teaching neonatal CUS interpretation considerably improved trainees’ knowledge and enhanced their skills in interpreting neonatal CUS.

## Background

Cranial Ultrasound screening is a very common, non-invasive imaging procedure used frequently in neonatal intensive care units (NICUs) to diagnose brain injury in full-term and preterm infants. Serial scans are performed routinely to assess the severity of ischemic and hemorrhagic lesions and monitor their progress [1]. In the United Kingdom and Australia, CUS’s interpretation is a significant and integral part of pediatric and neonatal specialties [2, 3]. In those countries, pediatricians and neonatologists are usually responsible for performing the scans and interpreting the results themselves, allowing them to correlate their findings with the patients’ clinical status and provide immediate intervention as needed. However, there is a lack of formal training in this area in Canada. The training objectives of the pediatric residents and neonatal-perinatal medicine (NPM) trainees specify that they should be able to “recommend or select appropriate diagnostic imaging” with no mandate for the acquisition of image interpretation skills [4, 5].

Currently, in all Canadian NICUs, when a neonatologist asks for CUS, it is performed by a trained sonographer and reported by one of the radiologists. This process usually takes hours to complete. Yet, in other clinical areas such as Emergency Medicine and Anesthesia, both hands-on ultrasound skills and image interpretation are part of the training. A recent survey done by the author, Ben Fadel N et al. (2019), indicated that NPM trainees unanimously agree that the learning of ultrasound skills offers many benefits when it comes to patient care, and acquiring them is very important for their future careers. However, there is a lack of formal curriculum for both hands-on ultrasound skills and image interpretation skills, both of which are urgently needed [6].

Having the skills to interpret neonatal CUS would allow physicians to comprehensively correlate clinical information with CUS images, leading to timely and improved patient care. The goals of the radiologists and neonatologists when it comes to interpreting a CUS are fundamentally different. Neonatologists look at answering a specific clinical question that narrows a clinician’s differentials and guides emergency treatment for conditions such as bleeding and progressive dilation of the ventricles. In contrast, radiologists are the health care’s imaging experts; they can provide more detailed information on complex cases with greater anatomic specificity or identify alternative diagnoses.

For visual subjects such as diagnostic images, WBL systems have been proven to make a suitable learning modality. They are increasingly used and readily available for teaching radiology residents in different areas such as mammography, fluoroscopy, interventional radiology, computed tomography, and ultrasound [7–9]. Schlorhaufer et al. (2012) described innovative, web-based, interactive polytrauma CT scan tutorials to support radiology residents’ training in writing a fully detailed CT scan report. The Web-based tools used to attain competence included: didactic concept teaching, normal/abnormal movie clips of normal and abnormal findings for comparison [10].

The learning process in health care is overly complicated due to the complexity of different clinical situations. Theoretical knowledge, communication, clinical and procedural skills are essential for clinical competence. Any training program should ensure that all trainees have the appropriate learning opportunities, knowing that learning in an acute care setting is quite different from learning in community medicine. Feng et al. (2013) discuss how knowledge and skills learned through clinical practice are the core to success and are fundamental to the quality of care provided [11]. However, such practices can be very unpredictable depending on placements, rotations and availability of patients. If effectively designed, implemented, and deployed, WBL could complement the busy trainees and practicing physicians’ clinical experience. The technology can also accommodate shift schedules and distance learning and can be easily expanded and modified [12–14]. WBL has been found to be as effective as other teaching modalities, with advantages such as availability, overcoming time and distance barriers, as well as fostering independence and collaboration [15, 16]. Other desired benefits of WBL include accessibility, hyperlink functions and the simplicity of content updates [17–19]. Based on studies indicating that WBL may be developed and implemented in medical settings with successful results, medical institutions are increasingly exploring web technology and the necessary support tools to maximize students’ learning experience [20].

Like any other education modality, the development of WBL needs systematic planning that allows for a more efficient acquisition of knowledge and skill, a process that is referred to in the literature as instructional design. Biggs (1999) described three essential factors in instructional design: (1) well-defined learning objectives, (2) instructional methods that help achieve the learning objectives, and (3) trainees’ assessment [21]. There are numerous models of systematic instructional design processes that have worked well for different WBL designers [22]. Some of the widely used models discussed in the literature include; ADDIE (Analysis, Design, Development, Implementation, and Evaluation); ASSURE (Analyze, state objectives, Select media, Use the media, Require participation and Evaluate); the Dick & Carey; Gagne’s Nine Events of Instructions; Hypermedia Design; Rapid Prototyping models and the SAM (Successive Approximation Model) [22–25]. Each model framework has its advantages and disadvantages. The designers’ choice of which model to use depends on their needs, resources available, and budget [24].

The principal objective of this study was to provide our trainees with the opportunity to learn how to interpret CUS studies performed by sonographers in a timely manner. We used the ADDIE instructional design model as a framework to plan, develop and evaluate a new WBL module on neonatal CUS interpretation. We choose the ADDIE model because of its simple step-by-step organized and thorough approach, as described below. Selecting this model worked well with the available resources and a limited budget; it allowed us to create a learner-centred and goal-oriented design geared towards a reliable measurement of trainees’ knowledge, skills and attitudes.

## Methods

### Design of the education intervention

We used the five phases of the ADDIE’s model to guide the module development, starting with identifying trainees’ needs, educational objectives, and preferred content delivery methods. We worked with an education technology specialist to design the WBL module, and this was followed by implementing the module and evaluating both the educational intervention and trainees’ performance. The Evaluation phase was completed in a very systematic process. We followed a pragmatic paradigm that uses qualitative and quantitative research methods and focuses on what works, unlike a more positivistic or interpretive approach to research that focuses on absolute truth or reality [26, 27]. This paradigm allows for the flexibility to choose various combinations of methods and to inquire from a variety of perspectives [28]. During this phase, the feedback gathered measured trainees’ satisfaction and identified what was working and what did not work. Even though the evaluation is the last phase of the ADDIE model, it is also the starting point of re-analysis and further design and development modifications.

### Needs analysis

By conducting a thoughtful and thorough needs assessment, assumptions regarding the project’s acceptance by trainees, trainees’ characteristics, including baseline knowledge, can be avoided. In this study, we used a needs assessment survey questionnaire to explore trainees’ existing background, their perceived level of comfort with the knowledge and the skills they needed for neonatal CUS interpretation, in addition to their learning needs and attitudes towards different learning methods suitable for gaining the required knowledge and skills. We also explored their expectations regarding module content that will enhance their learning experience. The design of the needs assessment questionnaire was informed by a thorough review of the literature to better understand the needs assessment models and methods of data collection, as described by Laidlaw et al. (1995); Hesketh & Laidlaw (2002) [29, 30]. The survey questionnaire consisted of 12 questions which asked the trainees to indicate the degree to which they agreed or disagreed with various statements on a 5-point Likert scale from strongly disagree to strongly agree. The last question was open-ended and focused on the use and development of educational activities. Two neonatologists and one radiologist reviewed a draft of the questionnaire for content validity. This review ensured that the contents were appropriate and relevant to the topic and that the questionnaire was not missing any pertinent items [31]. Following the survey questionnaire’s administration, we performed an internal consistency analysis based on the respondents’ data. Using Cronbach’s coefficient alpha, we demonstrated that each item belonged to the questionnaire, with an acceptable alpha value of 0.71 (CI −.51, 0.79).

All residents enrolled in the pediatrics training program, and all fellows enrolled in the NPM training program at the University of Ottawa in the 2016–2017 academic year were invited to participate in this study (*N* = 62). Including all trainees was essential to collect accurate data and to delve deep into trainees’ needs in this area of neonatal care. We obtained approval from a local ethics board prior to the initiation of the study. The questionnaire was distributed online through RedCap®, a secure web application for building and managing online surveys and databases.

The survey results showed that the majority of trainees were not entirely comfortable with their neonatal CUS interpretation skills. Also, they see these skills as vital parts of their training and, therefore worthy of greater attention. Trainees perceived most current teaching instructional modalities in this specific neonatal area as minimally effective; however, WBL modules were considered a potentially convenient and effective way to learn.

### Module design and learning activities

With the information gathered from the needs assessment and input from two expert neonatologists and one radiologist, we were able to design a new WBL module that is well-aligned with trainees’ educational needs. The chosen design prioritized simplicity and clarity, with content that is very explicit and comprehensive. We used knowledge, skills and attitude categories to frame the learning objectives and align them with the required competencies. We selected the contents on the basis of what trainees need to know and need to be able to do to achieve the intended learning outcomes [8]. Trainees were expected to understand the neonatal brain ultrasound protocol, identify the anatomical landmarks, differentiate between a mature and premature brain on ultrasound images, and identify and diagnose pathological changes.

**Fig. 1:**
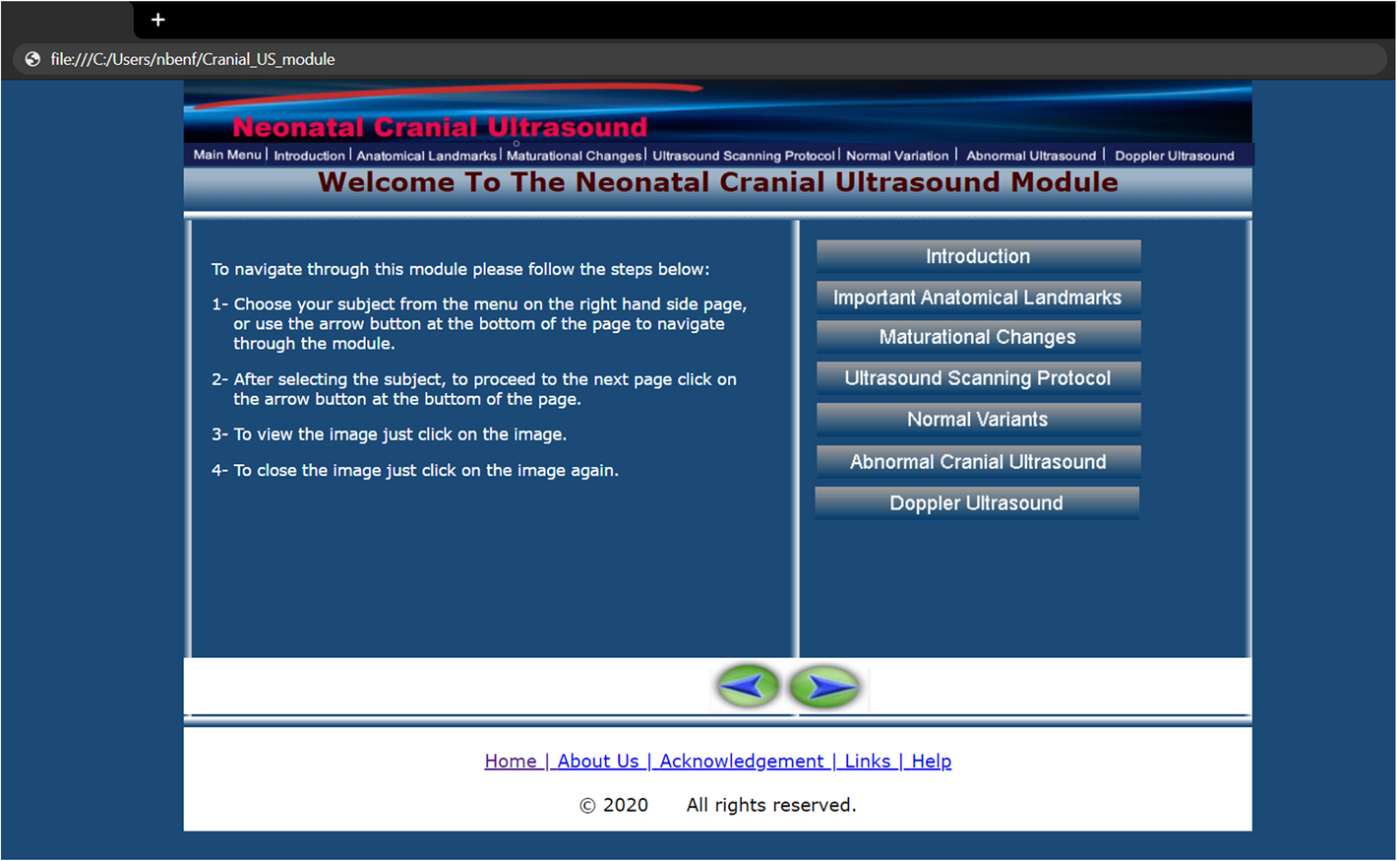
Screenshot of the module welcome page. Legend: The first page of the module shows the list of the seven chapters and instructions on how to navigate the module

**Fig. 2:**
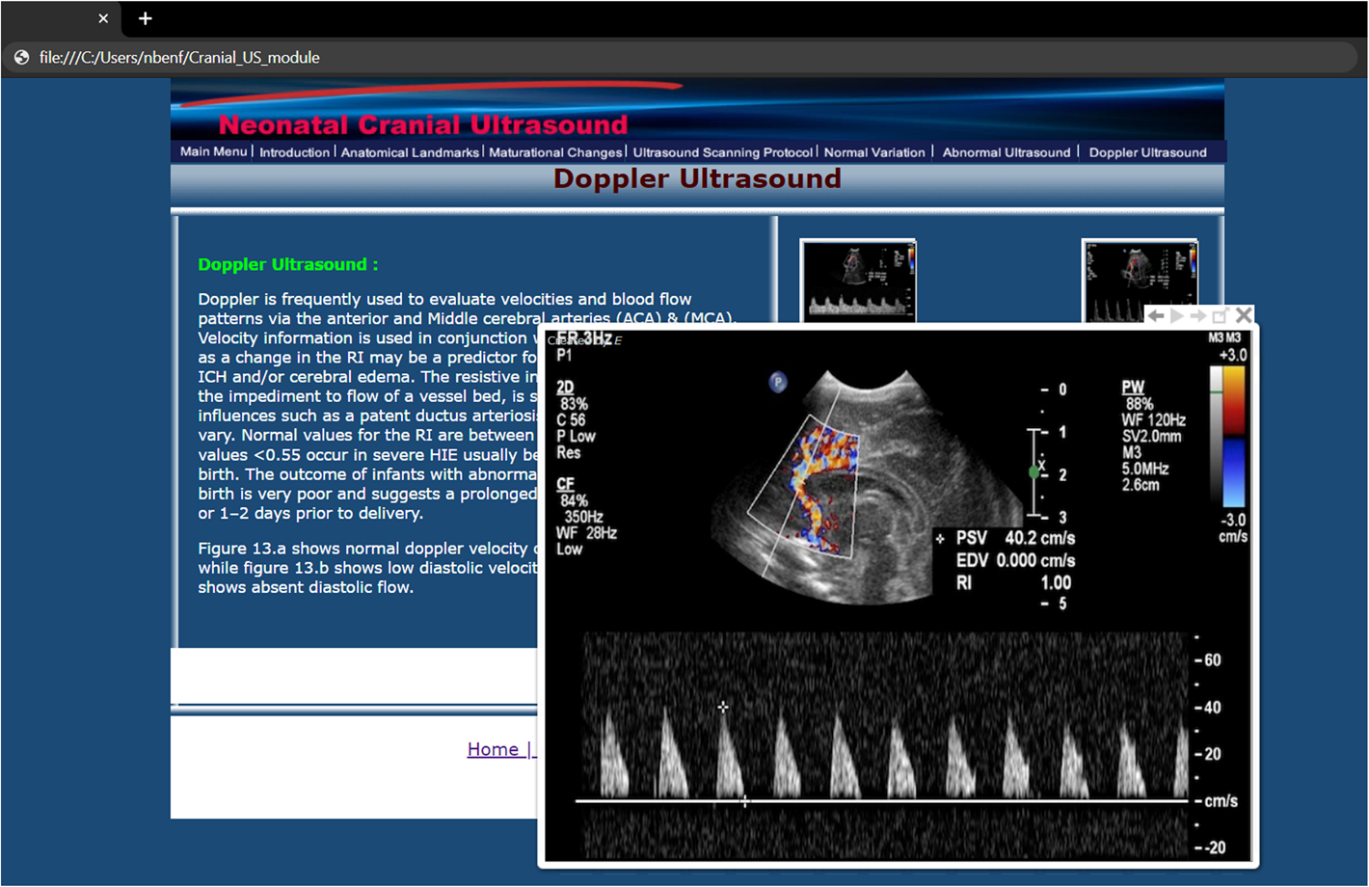
Screenshot of the Doppler CUS page Legend: The images are displayed as a thumbnail and will enlarge once the learner clicks on it. Enlarging more than one image allows for comparison

The module consisted of seven chapters, accessible through a navigation menu bar or sequentially through a forward button. It is composed of over 150 high-quality images, graphics, and text to enforce the learning experience. They are perfectly suited to help trainees learn about the various dimensions of focused neonatal CUS **(**Figs. 1 & 2**).** We selected specific learning activities that would lead to the achievement of the required outcome. Each chapter offers an end of chapter quiz to provide feedback regarding the trainees’ progress. In addition, the module offers an end of module quiz for assessing the overall knowledge gained. All quizzes contained answers to the questions displayed, which were only revealed when trainees decide to view them. For further detailed information about the subjects, we provided external resources through hyperlinks. The module includes multiple topics and subtopics. Moreover, learning of the material was designed to allow trainees to go through the content at their own pace, in an organized, step-by-step manner. Trainees were able to progress through a wide range of complexity levels as they worked to achieve a more in-depth understanding of other specific areas.

### Module development

We worked with an education technology specialist from CATmedia® (Local Web Design Company) to ensure that the interactive WBL methods and design were of high quality. Tools used to develop this novel module were Adobe® Suite CS6, HTML, CSS3 and Javascript. The module runs on any operating system platform (Microsoft Windows®, Apple®, Unix®, or Linux®) with any web browser (Internet Explorer®, Firefox®, Chrome® or Safari®). We acquired the anonymized ultrasound images from the hospital’s PACS system (picture archiving and communication system). We then imported them into Adobe Photoshop CS6 for further processing, such as removing personal data and re-labelling.

### Implementation

We housed the WBL module on the Children Hospital of Eastern Ontario’s (CHEO) Intranet site, password-protected, and was available to the trainees through the Department of Paediatrics’ internal webpage. The study duration was 6 months, after which the module became available to trainees. Only participants doing a rotation in the NICU were able to access the module for that period. After the first 6 months, the module was available 24/7 to all pediatrics and NPM trainees through CHEO’s intranet site access.

### Planning students’ assessment and module evaluation

We structured the evaluation process around Kirkpatrick’s Levels of evaluation (trainees’ reaction to the learning experience (level 1), knowledge and skills assessment (level 2), clinical behaviour changes (level 3) and overall impact on patient outcome (level 4) [32]. The Kirkpatrick evaluation model has been used for training program evaluation for more than 50 years. It provides a multi-level approach to evaluate training programs and assess trainees’ knowledge, skills, and attitude [33]. Exploring trainees’ reactions and satisfaction was a crucial step towards updating and improving the module’s delivery. Additionally, assessing the knowledge and testing skills gained by trainees after studying the module was an essential step to measure whether trainees have achieved the intended learning outcomes [33, 34].

We conducted the evaluation in four steps:

1. Trainees’ reaction to and satisfaction with the WBL module, using a satisfaction survey.
2. Knowledge assessment using a pre- and post-test.
3. Performance assessment using a pre- and post-test.
4. Behaviour change, using an impact questionnaire approach.

All study instruments were peer-reviewed by three experts in the field, which provided evidence of consistency and reliability.

#### Participants

**Fig. 3:**
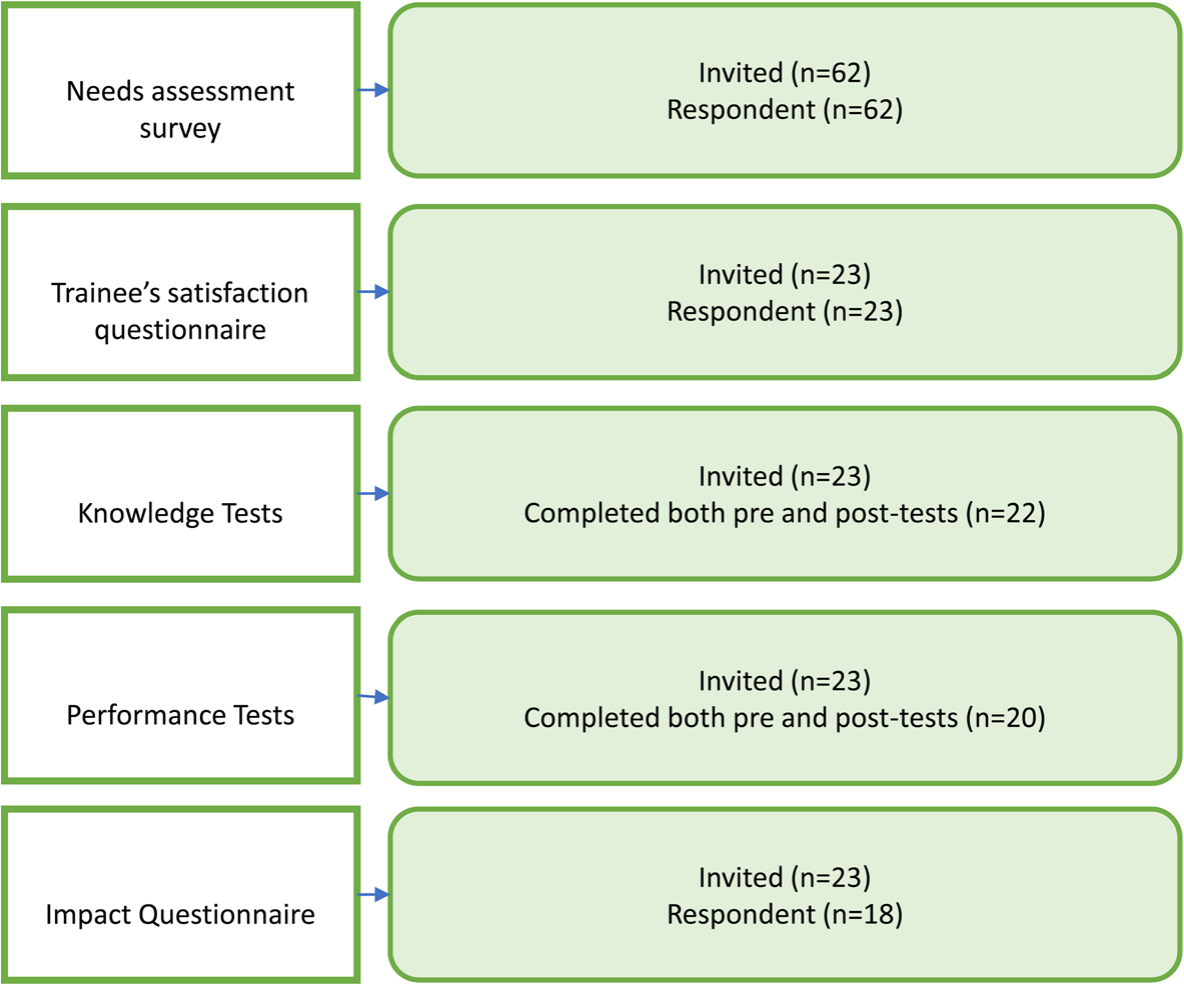
Study participants. All trainees in the pediatrics and NPM programs were invited to participate in the needs assessment. A convenient sample of 23 trainees rotating through the NICU in six months period was invited to participate in the evaluation process

All trainees (*n* = 23) scheduled for NICU rotations between July 2016 and January 2017 at the Children Hospital of Eastern Ontario (CHEO) were invited to participate in the four steps of this study (convenient sample). It was not feasible to include all 62 trainees in this part of the study, given the resources and time required (Fig. 3). Participants included 13 pediatric residents (3 first years, 3 s years, 4 third years, 3 fourth years) and 10 NPM fellows (1 first year, 6 s years, 3 third years). We included trainees at different training levels because the interpretation of neonatal CUS is a stand-alone skill that does not involve increased concept complexity, and it can be learned at any stage of training.

#### Ethical considerations

There were no ethical concerns for this study. Ethical approval from the local ethics board was obtained prior to the initiation of the study. All trainees rotating through the NICU over the 6 months had an equal chance to participate; training status or educational experience did not affect participant eligibility. Participation was voluntary, and trainees were asked to sign a consent form before the study’s commencement. Each participant had a unique ID to use for pre-tests and post-tests. All study material and test results were kept strictly anonymous and confidential.

### Evaluation steps

#### Step 1: trainees’ satisfaction

In this step, we focused on assessing how trainees reacted favourably to the educational intervention and explored their views on navigation through the WBL module, usability, content, and design. We also assessed whether the proposed learning objectives were met. We used a modified version of the satisfaction questionnaire by Larsen et al. (1979), with some elements adapted from the questionnaire used by MacDonald et al. (2002) [35, 36]. The survey was then hosted on the online survey software program FluidSurveys™, and an invitation via email was sent to all trainees who participated in studying the module (*n* = 23). All trainees who participated in the study and reviewed the module (*n* = 23) completed the survey which was submitted anonymously by the participants within 1 week of finishing the NICU rotation.

The survey sought feedback on the extent to which content met respondents’ learning needs, the relevance and effectiveness in achieving the learning objectives, technical aspects and appropriateness of format, the most helpful aspects, and suggestions for improvement. We asked trainees to indicate the degree to which they agreed or disagreed with various statements regarding the module on a 5-point Likert scale, with 1 meaning they strongly disagreed; 4 meaning they strongly agreed, and 5 for “don’t know” a category included to minimize the number of missing responses. We also asked them to indicate any aspects of the module they found helpful and suggestions for improvements. We summarized trainees’ demographics using descriptive statistics and used frequencies and percentages to summarize trainees’ satisfaction with the module. We defined positive responses as (agree/strongly agree) and negative responses (disagree/strongly disagree). We also analyzed open-ended comments for recurrent themes. Two neonatologists and a nurse practitioner pilot-tested the survey to assess the wording and clarity of content and time needed to complete it. Once again, we calculated Cronbach’ ‘s Alpha after administering the survey and found an internal consistency of 0.68 (CI 0.41, 0.82). While the estimated alpha value was not high, it had considerable uncertainty, as reflected in the 95% confidence intervals. However, as Tavakol et al., (2011) suggest, the number of test items in this survey is small, and Cronbach’ ‘s Alpha will, thus, underestimate the internal consistency [37].

#### Step 2: knowledge assessment

This evaluation step examined whether trainees learned what they were taught and the extent to which the WBL module changed their knowledge about neonatal CUS. The assessment aimed to provide objective evidence that learning has taken place through a simple one-group pre-and post-test design. We developed two knowledge tests with questions of comparable difficulty. We did not use the same test items for the pre-and post-tests to avoid familiarity with the questions, which may improve trainees’ knowledge. We based test items specifically on the learning objectives defined for each section of the module. We included a mix of multiple-choice questions with four response options and short answer questions with ultrasound images and Doppler tracing. We included only questions to which we provided clear answers in the module. We limited each test to 12 items, and both the pre-and post-tests had the same number of multiple-choice and short answer questions that trainees could answer within the allotted 20 min.

The difficulty in both tests was highly comparable. Each question represented similar findings (i.e., the question about Doppler tracing in the pre-test shows decreased resistive index of the middle cerebral artery). In the post-test, it shows an increased resistive index of the same artery). With one of the radiologists’ help, we created a model answer key to ensure reliable and transparent scoring and limit the risk of inconsistent assessment.

For the validation of the knowledge tests, we adapted the rigours process described by Moore et al., (2017) [38]. A team of three content experts (two neonatologists and one radiologist), who were not involved in creating the tests, reviewed the drafts of the tests. They provided feedback on the content and image quality’s appropriateness and relevance. They also completed question-objective matching to ensure construct validity. Two neonatologists and a nurse practitioner then pilot-tested the tests, and we incorporated their valuable feedback into the final version. Although Cronbach’s Alpha was estimated at the lower side 0.64 (CI 0.38, 0.82) for the pre-test and 0.59 (CI 0.28, 0.79) for the post-test, it might have underestimated the internal consistency because of the small number of test items.

We gave the knowledge pre-test to individual trainees at the beginning of the NICU rotation. A research assistant observed trainees during the test and collected the answer sheets. Once the trainees completed the pre-test, they were given access to the module for their four-week NICU rotation. One to 2 weeks after the rotation, they carried out the post-test but did not have access to the module at that time. Participants served as their own controls, and pre-and post-test data were collected and compared. We included only participants who completed both tests in the analysis of the results (*n* = 22). One trainee was not available to write the post-test.

Trainees had to indicate their training level and used the same ID code on pre-tests and post-tests to allow for comparison. For both pre-and post-tests, the maximum score was 15. Using the software SPSS version 22.0 (SPSS Inc., Chicago, IL, United States), we summarized test scores and computed descriptive statistics (i.e., mean and standard deviation). We compared overall scores using a paired t-test considering *P*-values less than 0.05 to be statistically significant. We also compared individual scores to other participants to determine if there were any differences or trends in knowledge gained from the module between junior and senior residents.

#### Step 3: performance assessment

This part of the evaluation aimed to assess the extent to which trainees put their acquired knowledge into practice and whether the module influenced their behaviours/skills change. We used pre-and post-performance tests to assess the skills of CUS interpretation.

In consultation with our radiologist, we downloaded two sets of neonatal CUS studies from the hospital PACS. Each set contained ten studies, and each study consisted of 20–30 de-identified ultrasound images studying all anatomical areas of the neonatal brain. The two tests were comparable; each had similar diagnoses or a different entity of the same disease. For example, CUS study #1 in pre-test and CUS study #1 in the post-test had the diagnosis of periventricular echogenicity and periventricular leukomalacia, respectively. The ultrasound study in the pre-test represented the mild form, while the study in the post-test represented the most severe form of the same pathological process.

We created a specific answer sheet, similar to radiologists’ reporting templates for the neonatal CUS. The trainees’ task was to review the studies and write a brief interpretation of the findings seen on 12 anatomical areas of the neonatal brain (normal and abnormal) and then write down the final diagnosis. Trainees reported their findings on the template for every study, and we used the formal radiologist report as the model answer key. All studies were reviewed by one radiologist and one neonatologist to ensure image quality, appropriateness, relevance and meeting the learning objectives. We were not able to assess internal consistency using Cronbach’s Alpha since our sample size and the test items numbers were too small. However, we had two neonatologists and one nurse practitioner pilot test the pre-and-post practical test and the reporting template. They all reported the studies to be clear with excellent image qualities and the reporting templates were very easy to use.

Early in their NICU rotation and before studying the module, trainees (*n* = 23) were able to access the first 10 CUS studies for the performance pre-test. Trainees completed this test within 24 h of taking the knowledge pre-test. Trainees were also able to access the module immediately after finishing the performance pre-test. Trainees reviewed the module as many times as they needed during the 4-week rotation; however, the time they spent studying the module was not recorded because they had studied in their free time. One to 2 weeks after completing the NICU rotation, and within 24 h of taking the knowledge post-test, trainees were asked to take the post-performance test reporting the findings on the second set of CUS imaging studies. Trainees did not have access to the module content when they completed the post-test.

The final scoring of both pre- and post-performance tests depended on whether the trainee identified all pathology/abnormalities. The tests were scored “complete,” “incomplete,” or “wrong interpretation.” We assigned the incomplete category if the trainee did not identify all abnormalities. Using the final radiologist report as a model answer key, two trained neonatologist compared trainees’ answer sheets (CUS interpretations) to those of the radiologist and assigned a final score. Trainees used the same ID code on pre-tests and post-tests to allow for comparison. Similar to the knowledge tests, participants (*n* = 3) served as their own controls, and we included only trainees who completed both pre- and post-performance tests in the analysis of the results (*n* = 20). Three trainees did not complete one of the tests, either the pre-test or post-test.

#### Step 4: impact on trainees and behaviour change

Kirkpatrick et al. (2006) suggested that impact evaluations could help verify the link between learning and changes in trainees’ performance when combined with knowledge and skills evaluations [32]. Recognizing that assessing the impact on patients’ care and their outcome is very challenging due to multiple confounders, we looked only at the overall perceived impact that the module had beyond the trainees’ satisfaction and whether trainees had applied their newly gained knowledge and skills in the workplace [32].

One year after we closed the study, we contacted trainees involved in the original study (*n* = 23) and invited them to complete a short and focused anonymous impact questionnaire. We created the questionnaire with guidance from Kirkpatrick’s description of Level 3 evaluation. The questionnaire consisted of six multiple-choice questions and two open-ended questions to determine the extent to which trainees used their previously acquired knowledge and skills in practice. We also solicited additional comments about the module. Responses were confidential, and participation was voluntary. Similar to the other study instruments, this short questionnaire was validated locally. Two senior NPM fellows and one neonatal nurse practitioner reviewed it and provided feedback on the preliminary questionnaires’ appropriateness, clarity, and feasibility. Necessary revisions were made before the final version was sent out. At the time of sending this questionnaire, ten participants were finished with their training and assumed faculty positions. One trainee joined another subspeciality training program.

## Results

Analysis of the satisfaction questionnaire showed that all respondents agreed/strongly agreed that the quality of training they received from the module was excellent. Additionally, all respondents agreed they received optimal training and were very satisfied. All respondents indicated they would definitely recommend the module to any colleague interested in learning how to interpret neonatal CUS. Ninety-one percent of respondents strongly agreed that the module contents were relevant to their practice. Ninety-six percent strongly agreed that the module provided appropriate technology use with a reasonably fast downloading speed and display of images, aesthetically pleasing graphics, uncluttered presentation, and clearly labeled anatomical landmarks.

On a scale of one to five, trainees rated their knowledge and skills in interpreting neonatal CUS before studying the module at a Mean value of 1.52 (SD 0.79) compared to after studying the module with a Mean value of 3.65 (SD 0.65). Ninety-one percent of respondents said they would use their acquired skills to interpret neonatal CUS before viewing the radiologist’s report. Ninety percent of trainees indicated that the time allocated to complete the module should not be longer than 90 min. When asked about what aspects of the module they found particularly helpful, responses were very positive about the module’s value to their learning. They provided insightful comments and suggestions for improving the module. Among other things, the trainees said all chapters in the module were easy to read and follow; images and illustrations were very clear and useful; it was well organized and easy to navigate, and the information was clear and relevant. Suggestions to improve the module included adding live scanning videos, more flow Doppler tracing, adding more information about the long-term outcomes of different pathologies, and adding more case-based questions.

Additionally, some interesting points emerged from the open-ended questions. Firstly, the trainees’ expressed willingness to learn the neonatal CUS interpretation and perform the ultrasound procedure themselves. Secondly, there was the suggestion to convert the module into an online application (app) that they can access from a smartphone or tablet at any time.

The Mean baseline knowledge test score (pre-test) was 6.23 out of a total score of 15 (42%). The mean score post-test was 11.34 out of 15 (76%). The change in score from pre-test to post-test was positive and statistically significant (*p* < 0.001) (Table 1).

**Table 1:** Paired comparisons of overall total scores between pre & post knowledge tests

	N	Pre-test	Post-test	Mean change post-pre	95%CI	*p*-value*
		Mean ± SD	Mean ± SD	Mean ± SD.		
Overall Total Scores (15/15)	22	6.23 ± 2.80 (42%)	11.34 ± 1.71 (76%)	5.11 ± 2.45	4.02, 6.20	< 0.001*

Legend: The change in scores from pre-test to post-test was positive (34%) and statistically significant (*p* < 0.001)

*P < 0.05. ^ Paired t-test. Adjusted *p*-value using Holm’s method

**Fig. 4:**
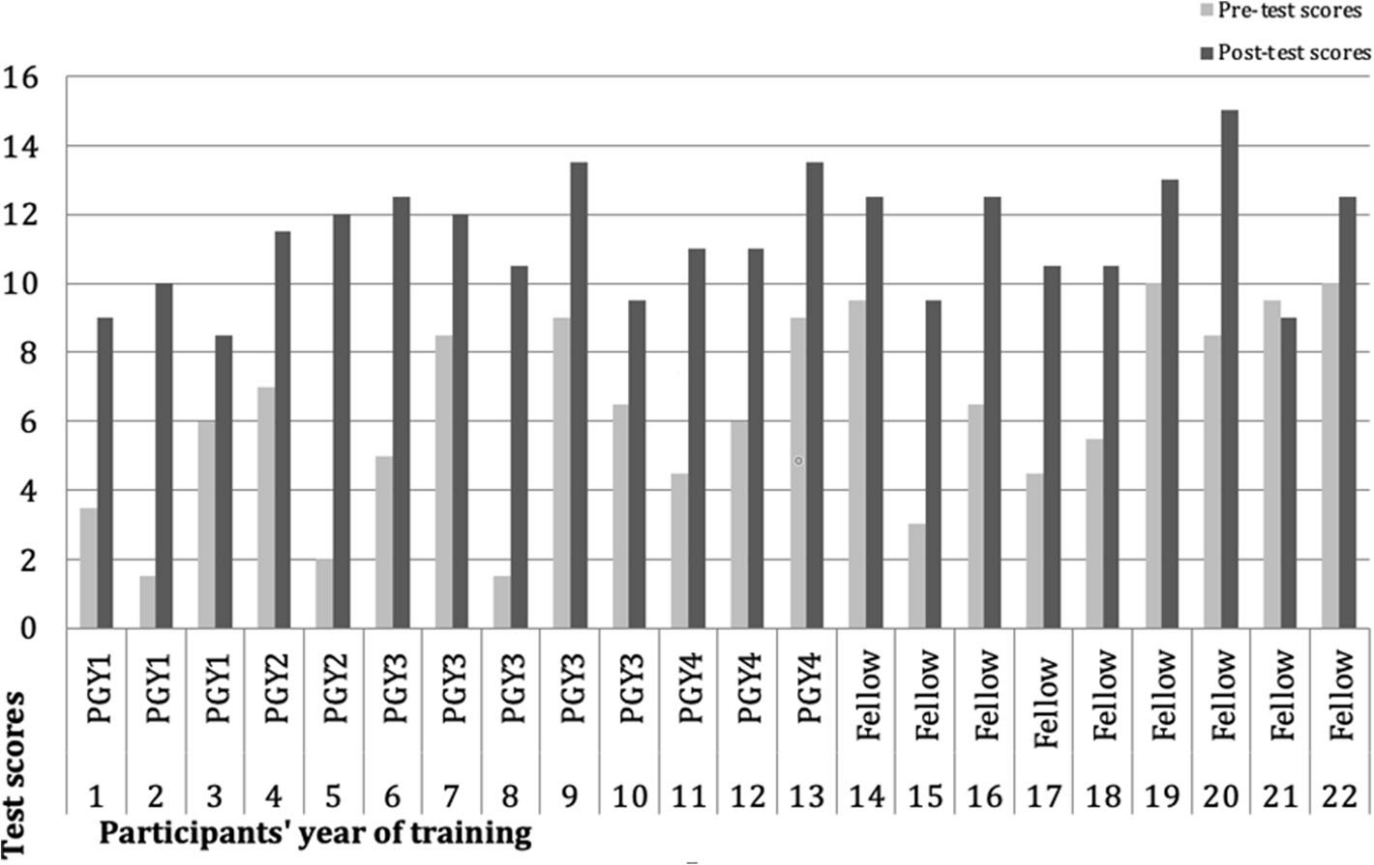
Pre- and post- knowledge test scores by for each participant. Legend: Shows individual trainees’ baseline knowledge and improvement in post-test scores

Figure 4 shows the individual trainees’ pre-test and post-test scores. Almost all the trainees had variable degrees of knowledge gain. Looking at baseline knowledge levels among trainees’ subgroups, the Mean pre-test scores increased with advanced training level, indicating that senior trainees are gaining some neonatal CUS knowledge throughout their training.

For the performance tests, each trainee submitted 10 answer sheets for the pre-test and 10 answer sheets for the post-test (one interpretation sheet for each CUS study). In total, we collected 200 pre-test answer sheets and 200 post-test answer sheets from all trainees who completed both tests (*n* = 20). While 99 out of 200 answers were incorrect in the pre-test (49%), only 17 of 200 had an incorrect diagnosis in the post-test (8%) (*p* < 0.001). There were only 52 (26%) complete answers in the pre-test compared to 126 (63%) in the post-test (*p* < 0.001).

**Fig. 5:**
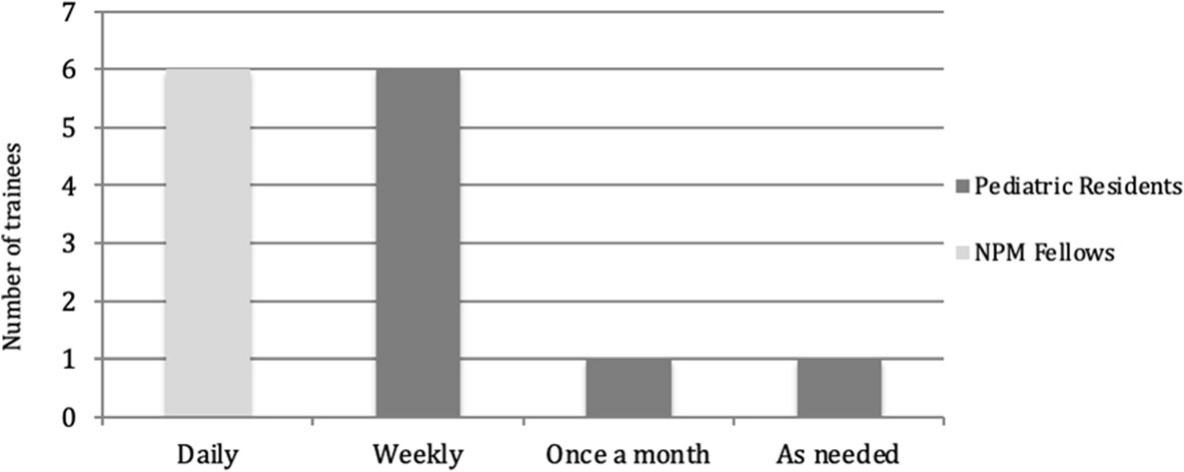
CUS knowledge and skills use by trainee type after they studied module. NPM fellows kept using the learned skills daily while most pediatrics residents used their skills weekly

Out of the invited 23 trainees, 18 trainees responded to the impact questionnaire (78%). Thirty-three percent were NPM fellows (*n* = 6), and the rest were pediatric residents. Seventy-eight percent of respondents (*n* = 14) said they had used their acquired knowledge during the previous year. Among those, all fellows applied the knowledge on a daily basis, while most pediatric residents used it on a weekly basis (Fig. 5). Four pediatrics residents indicated they had not used their skills because they sub-specialized in another area or worked in hospitals that did not do CUS.

Sixty-seven percent of respondents indicated that the overall training they received was very beneficial to their work. All respondents stated that they shared their knowledge and skills with medical students and junior trainees.

A selection of answers to the open-ended question (How did the knowledge and skills you learned benefit you on the job?) include:

- *“The module was my only source of learning about Neonatal CUS.”*
- *“I started teaching all juniors who come through NICU how to interpret CUS study.”*
- *“I reviewed each study before I read the radiologist interpretation.”*
- *“I was able to assure many parents immediately that their baby’s CUS is normal and always felt good.”*
- *“Every time I saw abnormalities in the CUS study, I called the radiologist and asked for the formal interpretation so that the diagnosis could be discussed with the family in a timely fashion.”*

When asked to provide recommendation and further comments on the module, trainees wrote: “would have liked to have the module earlier in training, should be made mandatory to PGY1 residents; would like a similar module for magnetic resonance images (MRIs); hands-on training on how to perform the procedure of CUS not just learn the interpretation of the images”. One trainee wrote, “Even though I am doing endocrinology but still grateful that I had this learning opportunity, it made it easier for me to understand the radiology reports.”

## Discussion

Despite the importance of learning the basic skills of neonatal CUS interpretation, a thorough search of the relevant literature did not yield any studies that describe neonatologists and pediatricians’ needs in this area of North America. To the best of our knowledge, this was the first study to recognize the learning needs of trainees in this vital area of neonatal care and the first study to create an effective WBL module based on those needs. The module contributed to trainees’ self-directed learning opportunities. It helped them achieve competency in interpreting neonatal CUS, which may lead to better patient satisfaction if the knowledge and skills are appropriately and consistently applied in practice.

Previous research work has shown that regardless of the pedagogical approach to diagnostic imaging education, image interpretation is a complex perceptual and cognitive process that requires integrating many knowledge and skills [39, 40]. Van der Gijp et al. (2018), summarized the knowledge and skills involved in radiological image interpretation using a framework that included three components: perception, analysis, and synthesis. This framework describes the cognitive process of initial recognition of normal and abnormal findings, then distinguishing relevant from irrelevant findings, followed by integrating findings and formulation of the final diagnosis [41]. We find this framework especially useful in guiding module development and pre-post-test blueprint preparation for trainees’ assessment. The WBL module provides relevant knowledge of anatomy and pathology, and without this prerequisite knowledge, learning the skills of CUS image recognition and interpretation would have been ineffective. The pre and post-tests assessed trainees’ abilities to recognize images, detect pathology and come up with a full diagnosis.

Feedback on the module was overwhelmingly positive, and perceptions of the difficulty of the topic decreased significantly. They provided excellent suggestions and asked to incorporate videos, Doppler tracing, and more cases with discussions around patients’ outcomes concerning different CUS pathology. These were reasonable changes that were made when the module was updated.

Similarly, their request to convert the module into a mobile application and introducing hands-on training for CUS, a rapidly growing skill in the critical care setting, was very intriguing. Recently, a few medical schools in Canada and the US have started incorporating hands-on ultrasound skills teaching in their undergrad curricula. This integration is indeed part of our postgraduate training program’s future teaching and research plans.

We recognize that even though trainees’ reaction was very positive, it is limited by its reliance on perception and is highly subjective, as trainees may believe that they have learned effectively when, in fact, they may not have. Roh et al. (2014), argued that learners’ satisfaction could be very subjective. Still, it is essential in any training evaluation as they are the primary stakeholder, whose satisfaction is one of the important quality indicators of teaching [42]. While skill assessment is preferable to demonstrate improvement in learning, Cohen et al. (1981) and Seymour et al. (2000) found that students’ perception of their learning correlates highly with scores on achievement tests [43, 44]. As such, a negative reactions may reduce motivation and the chance of learning if trainees do not react favorably [30]. Amin and Khoo (2009) discussed how trainees’ satisfaction does not always mean that knowledge and skills have improved [45]. However, improvement in these areas increases the probability of a positive impact and behavioural change [32]. Correlating the achievement test scores with the individual trainees’ satisfaction and learning would have added more validation to our study results; however, this step was not possible because of the complete anonymity of the satisfaction survey. Nevertheless, considering that the WBL module was the first and the only formal learning material put together to teach neonatal CUS interpretation, it is still possible that this has influenced trainees’ satisfaction as they did not have any other learning modality to compare to the module.

There was a 34% increase in knowledge tests scores. The 95% CI was relatively narrow between 4.02 and 6.20; therefore, we can consider the difference in test scores to be practically important, not just statistically significant. Bearing in mind the years of experience, senior residents and senior fellows had better pre-test scores and baseline knowledge than their junior colleagues, as shown in Fig. 4**,** indicating that they had benefited, to some degree, from informal CUS training over the years. Therefore, introducing this module earlier in training years would benefit junior trainees and advance their skills, as suggested by some of them.

Performance assessment with practical tests provided objective proof to support the WBL module’s effectiveness and knowledge transfer, similar to the conclusions from the knowledge test scores. We are aware that measurement of a performance change is more challenging to assess than knowledge gain, and that change may not have happened solely from learning from the module. Performance change could have occurred from some uncontrolled factors, which can be very difficult to isolate [46]. Trainees who had the pre-test and studied the module might have been more motivated in attending informal teaching, and their performance change could have also been due to the passage of time as the trainee progressed through their rotations. Having a control group could have eliminated this, but that was not possible due to the small sample size. Nevertheless, the results of this part of the trainees’ assessment complemented the positive results from the knowledge tests.

While a follow-up test would give a more objective evaluation of knowledge retention than self-reported satisfaction, research shows that clinical skills retention may decline within 3 months after training if the skills are not actually applied and used [47]. However, deliberate practice is the key to master the skills and maintain competency [48]. In this study, most of the trainees kept using the skills on the job and contributed to teaching juniors to help them gain competency in CUS interpretation early on in training. Most importantly, they felt more confident to speak with parents about their babies’ CUS results in a timely fashion, assuring them when the results were normal and provided support when results were abnormal. This knowledge transfer is the ultimate goal of this educational intervention and is objective evidence that learning has occurred. It is the expectation that all trainees discuss their findings with the consultant before approaching patients with critical results, which further enhances their learning.

In this study, we have not assessed the impact of learning on patient care. We could have surveyed the families regarding the value of immediate CUS interpretation, or hospital stays could be compared before vs after the program to see if outcomes were actually affected. These areas can provide the basis for future research.

Many studies have shown that evaluating educational interventions using a multistep evaluation process allows for understanding how effective the intervention is and how it can be improved [32]. Evaluating the implementation and the application process is just as important to ensure an effective outcome. Findings such as behavioural change, combined with high trainees’ satisfaction and knowledge gain, are indicators of high intervention effectiveness and proper implementation.

### Study strengths

Choosing ADDIE’s multistep approach for this project allowed us to create a learner-centred, goal-oriented design geared towards a reliable measurement of competencies in the interpretation of Neonatal CUS. The process was systematic and thorough, yet relatively simple, with clear aims and objectives. Most importantly, it worked well with the available resources and budget. In addition, conducting a multi-level evaluation based on Kirkpatrick’s evaluation model strengthened the results and led to concrete conclusions. While the traditional pre/post-test method has limitations, the use of the two types of achievement tests (knowledge and performance assessment) complimented our findings. Repeated retrieval of facts and concepts might have improved the ability to apply knowledge in practice, echoing the work done by Butler (2010), who showed that testing that allows learners to practice could facilitate the application of knowledge [49].

### Study limitations

Following an experimental design using a control group was not appropriate in this study because of the small convenience sample of 23 trainees only. Using a control group would have helped guard against threats to internal validity. However, the educational experience of the control group could have been adversely affected. Also, while Kirkpatrick’s model is appropriate for evaluating learning, many exogenous factors may arise and influence the results with time. In this study, there could have been confounding variables besides the module that may have contributed to the outcomes. These variables could potentially be the trainees’ ability to learn, their level of training, their motivation, as well as possible exposure to another intervention such as informal bedside teaching during the study. We did not have information on how many times the individual trainees reviewed the module before taking the post-tests; this might also have improved some trainees’ scores.

Furthermore, we sent the reaction survey and post-tests at the end of the rotation, which might have resulted in a recall bias due to the time gap between completing the module and the end of the NICU rotation. Moreover, trainees’ reactions to the module might have been biased by the reaction to the application of the knowledge gained from the module during the NICU rotation. However, this may strengthen the argument that the WBL module was well perceived. Thus, trainees made their opinion on the module not only after studying it online but also after having the chance to put the knowledge they gained into practice. While immediate testing showed a positive impact on trainees learning, a delayed assessment of trainees’ knowledge and skills could have strengthened this study; however, this was not possible due to many logistical factors; many trainees moved to other hospitals and some have already graduated from pediatrics and neonatology.

## Conclusion

The use of a well-designed and implemented WBL resource for interpreting neonatal CUS has objectively improved trainees’ understanding of the topic and their ability to put their knowledge into practice. However, for trainees to gain expertise in those skills and maintain competency, they must keep practicing the skills and attending relevant continued medical education sessions (CME) to enhance their knowledge and expertise. Only then can they make a positive impact on patient outcomes and maintain patient safety.

Since the various neonatal brain pathologies that a physician practicing in NICU needs to recognize are well-known, it would be interesting for future research to assess the number of CUS studies that a trainee must interpret to reach and maintain competency in this area. Such research would be essential to determine the future role of this skill set in clinical practice, as well as the impact on patient outcome. With medical education taking a big step away from traditional classroom teaching, WBL modules would increase the availability of learning experiences for prospective trainees. The experience from this module has inspired the planning for similar modules of other topics with high-perceived levels of difficulty in different areas of neonatal medicine, such as neonatal ventilation and neonatal hemodynamics.

## Abbreviations

CHEO: Children Hospital of Eastern Ontario
CUS: Cranial Ultrasound
NICU: Neonatal Intensive Care Unit
NPM: Neonatal Perinatal Medicine
WBL: Web-Based Learning
PGY: Postgraduate year
PACS: Picture Archiving and Communication System
CME: Continued Medical Education
